# The Safety and Efficacy of Microbial Ecosystem Therapeutic-2 in People With Major Depression: Protocol for a Phase 2, Double-Blind, Placebo-Controlled Study

**DOI:** 10.2196/31439

**Published:** 2021-09-22

**Authors:** Arthi Chinna Meyyappan, Cassandra Sgarbossa, Gustavo Vazquez, David J Bond, Daniel J Müller, Roumen Milev

**Affiliations:** 1 Centre for Neuroscience Studies Queen's University Kingston, ON Canada; 2 Providence Care Hospital Kingston, ON Canada; 3 Department of Psychiatry Queen's University Kingston, ON Canada; 4 Centre for Addiction and Mental Health Toronto, ON Canada; 5 Department of Psychiatry & Behavioral Sciences University of Minnesota Medical School Minneapolis, MN United States; 6 Department of Psychiatry University of Toronto Toronto, ON Canada; 7 Department of Psychology Queen's University Kingston, ON Canada

**Keywords:** gut-brain axis, depression, microbiome, probiotics, fecal transplant, MET-2

## Abstract

**Background:**

The gut-brain axis is a bidirectional signaling pathway between the gastrointestinal tract and the brain; it is being studied because of its potential influence in mediating mood, anxiety, and other neuropsychiatric symptoms. Previous research examining the effects of gut microbiota on neuropsychiatric disorders suggests that gut repopulation treatments such as probiotics, microbe therapy, and fecal microbiota transplantation show promising results in treating symptoms of anxiety and depression. This study explores the use of an alternative gut repopulation treatment to fecal microbiota transplantation, known as Microbial Ecosystem Therapeutic (MET)-2, as an intervention against symptoms of depression. MET-2 is a daily, orally administered capsule containing 40 bacterial strains purified from a single healthy donor.

**Objective:**

The primary aim of this study is to assess changes in mood in people with major depression that occur pre-, post-, and during the administration of MET-2. The secondary aims are to assess changes in anxiety symptoms, blood biomarker concentrations, and the level of repopulation of healthy gut bacteria as a response to treatment.

**Methods:**

In this study, we will recruit 60 adults aged between 18 and 45 years old with major depression and randomly assign them to treatment or placebo groups. Patients in the treatment group will receive MET-2 once a day for 6 weeks, whereas patients in the placebo group will receive a matching placebo for 6 weeks. Participants will complete biweekly visits during the treatment period and a follow-up visit at 2 weeks post treatment. As a primary outcome measure, participants’ mood will be assessed using the Montgomery-Asberg Depression Rating Scale. Secondary outcome measures include changes in mood, anxiety, early stress, gastrointestinal symptoms, and tolerability of MET-2 treatment using a series of clinical scales and changes in blood markers, particularly immunoglobulins (Igs; IgA, IgG, and IgM) and inflammatory markers (C-reactive protein, tumor necrosis factor-α, transforming growth factor-β, interleukin-6, and interleukin-10). Changes in the relative abundance, diversity, and level of engraftment in fecal samples will be assessed using 16S rRNA sequencing. All data will be integrated to identify biomarkers that could indicate disease state or predict improvement in depressive symptoms in response to MET-2 treatment.

**Results:**

Given the association between the gut microbiome and depression, we hypothesized that participants receiving MET-2 would experience greater improvement in depressive symptoms than those receiving placebo owing to the recolonization of the gut microbiome with healthy bacteria modulating the gut-brain axis connection.

**Conclusions:**

This study is the first of its kind to evaluate the safety and efficacy of a microbial therapy such as MET-2 in comparison with placebo for major depressive disorder. We hope that this study will also reveal the potential capabilities of microbial therapies to treat other psychiatric illnesses and mood disorders.

**Trial Registration:**

ClinicalTrials.gov NCT04602715; https://clinicaltrials.gov/ct2/show/NCT04602715

**International Registered Report Identifier (IRRID):**

DERR1-10.2196/31439

## Introduction

### Background

Research efforts to identify the pathophysiology and underlying mechanisms of psychiatric illnesses, such as major depressive disorder (MDD), are on the rise owing to the staggering economic burden and prevalence of mental illness. In Canada, mental illness affects approximately 20% of people and costs the Canadian economy more than Can $51 (US $39.48) billion a year, of which MDD is a major contributor [1,2]. MDD is a highly prevalent and debilitating psychiatric disorder characterized by a persistent depressed mood and loss of interest or pleasure in the surroundings. Common symptoms of MDD can include but are not limited to depressed mood, anhedonia, changes in appetite, trouble sleeping, reduced energy and fatigue, feelings of worthlessness, guilt, hopelessness, and thoughts of death or suicide [3].

Although there are a variety of psychological, pharmacological, and neurostimulation treatment methods available for people with MDD, such as cognitive behavioral therapy, antidepressant medications, and electroconvulsive therapy for treatment-resistant cases [4-6], finding the optimal treatment method can be challenging owing to a high level of individual variability among patients. Variables such as environmental influences, stress, and genetics play a role in determining the severity and course of symptoms and the response to treatment [7-9]. Furthermore, emerging treatment options addressing individual variability in patients with MDD have demonstrated the potential for creating a more precise and personalized approach to treatment, specifically within psychiatry [10].

### Rationale for the Study

#### Composition of the Gut Microbiome

The colonization of bacteria in the gut begins at birth and significantly influences gut health throughout the lifespan [11]. In adults, the gut microbiome comprises more than 100 trillion commensal bacteria essential for the normal development and regulation of the immune system, central nervous system circuitry, and gastrointestinal (GI) functioning [12]. Under certain circumstances, environmental risk factors such as diet, lifestyle, stress, medications, and genetics can alter microbiota composition and deviate the homeostatic balance within the microbiome [13].

Previous studies have demonstrated that these alterations in microbiota composition owing to external factors may be mediated by the gut-brain axis (GBA). The GBA is a bidirectional, biochemical, and neural signaling pathway between the GI tract and the brain [14]. Although it has been long established that there is communication between the brain and the gut, only recently has there been a surge of interest in the GBA for its potential influence on depression and other symptoms of psychiatric illnesses [12]. Owing to the interaction between the gut and environmental risk factors of anxiety and depression, some interventions target the gut microbiome to aid in alleviating symptoms of anxiety and depression [15].

#### Gut Microbiota and Psychiatric Symptoms

Although alterations of gut microbiota may play a role in the etiology of psychiatric illnesses, the pathophysiology of MDD and how the GBA directly affects mood is complex. One of the most well-known hypotheses for the pathophysiology of depression is the monoamine hypothesis. It predicts that a deficiency in the levels of dopamine, serotonin, and norepinephrine in the central nervous system may be a key factor in the underlying mechanisms of depression [16]. Other central mechanisms that are also hypothesized to be involved in the pathophysiology of MDD include inflammation of the immune system and microbial imbalance [7].

Several studies have documented that patients diagnosed with MDD often present with elevated levels of proinflammatory cytokines, such as interleukin-6, and decreased levels of anti-inflammatory cytokines, such as interleukin-10 [7,17,18]. This proinflammatory state observed in patients with depression reaffirms the neuroinflammation hypothesis, which suggests that an increase in systemic inflammation is likely to be involved in the pathophysiology of depression by decreasing the synthesis and production of monoamines such as serotonin, resulting in a reduction of serotonin and an increase in depressive behavior [19].

Recent literature documenting the relationship between gut microbiota and symptoms of psychiatric illness has uncovered interesting findings. Microbial balance in the gut is an integral part of gut health; however, when one experiences an imbalance, the integrity of the protective epithelial and mucosal gut barrier can be compromised. This compromise might result in increased intestinal permeability of the GI tract, referred to as *leaky gut*. Leaky gut has been associated with various conditions, such as irritable bowel syndrome, autoimmune disorders, and multiple psychiatric illnesses [15]. Maes et al [20] demonstrated that bacterial translocation from the gut, also known as leaky gut, could activate immune cells to evoke specific IgA and IgM responses. Interestingly, patients diagnosed with depression often exhibit the same elevated levels of IgA and IgM responses, which may be owing to the progressive amplification of the immune pathways and overall increased systematic inflammation [20].

Despite these relevant advances in recent literature, a complete understanding of microbial changes in the gut and their associated outcomes is still required. Previous studies have demonstrated that patients diagnosed with depression often display altered gut microbiota composition compared with healthy controls [12,21,22], which may result from the decreased abundance and lack of diversity among healthy gut microbiota [21]. In an attempt to address this problem, related research has turned to fecal microbiota transplantation (FMT) as a potential treatment option to improve symptoms of depression. FMT is a therapeutic approach that aims to repopulate the gut of an individual with fecal bacteria from a healthy donor via colonoscopy, nasogastric tube, or oral administration [23,24]. A potential advantage of discovering this link between symptoms of depression and microbiota in the gut is the greater accessibility and modifiability of microbiota compared with the human genome, giving microbial therapies a greater opportunity for aiding in the personalization of treatments for psychiatric illnesses [25]. Furthermore, given the adaptable nature of the microbiome, it may be a good representation of the individual’s history and could better explain the differences in the risk of illness, disease course, and response to treatment.

### Microbial Ecosystem Therapeutics

Microbial Ecosystem Therapeutic (MET)-2 is an oral, daily administered, novel treatment approach designed to repopulate the gut with microbiota from a healthy donor. MET-2 is a defined microbial community comprised 40 strains of lyophilized bacteria that are lab-grown and purified from a healthy 25-year-old donor’s stool. MET-2 was originally developed by researchers at Queen’s University and the University of Guelph to treat symptoms of early depression and help restore the normal gut flora. The original mixture contained pure cultures of intestinal bacteria, referred to as *Repoopulate* or MET-1, which was composed of 33 strains of bacteria that were chosen for their favorable safety profile [26]. This mixture was then refined, modified, and improved to 40 strains to create MET-2. Capsules were chosen instead of traditional FMT not only to increase acceptability to participants but also to allow for easier administration of the product for consecutive days rather than the use of raw fecal material administered via rectal suspension.

This study aims to evaluate the effects of MET-2 on symptoms of depression using pre- and posttreatment scores for overall depression and specific depressive symptoms. Our primary aim is to demonstrate the efficacy of MET-2 treatment, in comparison with placebo, on mood and related symptoms in participants with depression, using the Montgomery-Asberg Depression Rating Scale (MADRS) [27]. Those with at least a 50% reduction in MADRS scores will be considered successful responders to treatment. The secondary aims are to assess changes in anxiety symptoms, immune marker levels in response to MET-2 treatment, and the safety and tolerability of MET-2 treatment, and to evaluate any potential correlations between early life stress (eg, childhood emotional, physical, or sexual abuse history) and response to MET-2 treatment.

## Methods

### Study Design

This study is a phase 2, randomized, double-blinded, placebo-controlled clinical trial exploring the efficacy of MET‑2 as a treatment for depressive symptoms in participants with MDD. We will randomize 60 participants with MDD into two arms: the treatment arm (30/60, 50%) and the placebo arm (30/60, 50%). Participants will consume either the investigational product MET-2 or a matching placebo daily for 6 weeks.

### Setting

This study will occur at 3 sites: the Providence Care Hospital, Kingston, Ontario, Canada; the Centre for Addiction and Mental Health, Toronto, Ontario, Canada; and the University of Minnesota Medical School, Minneapolis, Minnesota, United States. There will be a total of 6 visits: a screening visit, a baseline visit (week 0), 3 treatment-period visits (weeks 2, 4, and 6), and a 2-week follow-up visit as outlined in the schedule of assessments (Table S1 in Multimedia Appendix 1).

### Participants and Recruitment

This study will recruit 60 eligible participants aged between 18 and 45 years from the Kingston and Greater Toronto areas in Ontario, Canada, and the Minneapolis area in Minnesota, United States, using clinical referrals and web-based and paper advertisements. Web-based advertisements will be posted on social media platforms, whereas posters will be placed around university and college campuses, on community bulletin boards, clinics, and counseling centers. At the screening visit, all participants will be informed of the study, and any questions and concerns they may have will be addressed. Once consent is obtained, participants will then be thoroughly screened using inclusion and exclusion criteria (Textbox S1 in Multimedia Appendix 1) to ensure they are eligible for the study. Participants will be screened using the Mini-International Neuropsychiatric Interview to confirm the diagnosis of MDD and required a minimum MADRS score of ≥15 to be considered for inclusion in the trial.

### Treatment

#### Study Drug

The 0.5-g MET-2 and placebo will be supplied as capsules for oral administration. Placebo capsules will be filled with cellulose and will be identical in appearance. The participants will be provided with a 4-week supply of either MET-2 or placebo capsules at the baseline visit and a 2-week supply at the week 4 visit. Loading or booster doses will be kept separate from daily maintenance doses.

The 0.5-g MET-2 capsules are produced under conditions compatible with good manufacturing practices at the University of Guelph and are shipped at room temperature and sealed under anaerobic conditions. All capsules are to be stored at room temperature.

#### Administration of Study Drug

Participants who meet the inclusion criteria and pass screening will be scheduled to start treatment at baseline. At the baseline visit, all participants will receive a 10-capsule orally administered loading dose of 5 g of either MET-2 or placebo on both days 1 and 2 (Table S2 in Multimedia Appendix 1). The participant will consume the initial loading dose in the clinic and receive the second loading dose to be taken at home on day 2. The participant will also remain at the clinic for 30 minutes after receiving the first loading dose to ensure there are no adverse reactions to the medication. A 3-capsule maintenance dose containing 1.5 g of MET-2 or placebo will be administered for the remainder of the 2-week interval from 3 to 14 days. At the week 2 visit, participants will be asked to take a 10-capsule booster dose on days 15 and 16. The 3-capsule maintenance dose will be administered for the remainder of the study for a total of 6 weeks of treatment.

If a participant is a nonresponder (a participant whose MADRS total score is not reduced by at least 50% from baseline by week 4 visit), an additional 10-capsule booster dose is to be administered at week 4 for 2 days (days 29 and 30) followed by a maintenance dose of 3 capsules for the remainder of the study (Table S2 in Multimedia Appendix 1). Responders (participants whose MADRS score is reduced by ≥50%) will not receive a booster dose at week 4. Participants will not take the maintenance dose during the loading or booster dose periods.

#### Treatment Compliance

Compliance with treatment will be assessed by reviewing the participant’s personal logs and charts while documenting any missed and unused treatment material during the 6 weeks of intervention.

### Efficacy Endpoints

#### Primary Efficacy Endpoint

At the first endpoint, we will assess changes in symptoms of depression from baseline to week 6 as measured by MADRS scores.

#### Secondary Endpoints

The secondary endpoints are:

1. Changes in symptoms of depression from baseline to week 8 as measured by the Clinical Global Impressions (CGI), Snaith-Hamilton pleasure scale (SHAPS), and Quick Inventory of Depressive Symptomatology (QIDS)-SR16 scores.
2. Changes in symptoms of anxiety from baseline to week 8 as measured by the generalized anxiety disorder (GAD)-7 and Hamilton anxiety rating scale (HAM-A) scores.
3. Changes in immune marker concentrations from screening to week 4 and week 8.
4. Changes in symptoms of depression and anxiety from baseline to week 2, week 4, week 6, and the 2-week follow-up measured using the MADRS, HAM-A, and GAD-7 scores.
5. Changes in relative abundance, diversity, and level of engraftment in stool samples, as measured by 16S rRNA sequencing.

### Assessments of Safety and Efficacy

#### Primary Clinical Measures

Mood will primarily be assessed using the MADRS, a 10-item clinician-rated questionnaire used to evaluate the severity of depression.

#### Secondary Clinical Measures

Anxiety will primarily be assessed using the HAM-A, a clinician-rated questionnaire that measures the severity of anxiety symptoms. The GAD-7, a 7-item questionnaire, will be used to measure self-reported anxiety symptoms and severity. The CGI scale is a 2-item clinician-rated scale that will be used to assess the severity of illness and improvement of symptomology over time. The SHAPS is a 14-item self-rated instrument used to assess the presence of anhedonia. The QIDS-SR16 is a 16-item questionnaire that measures self-reported depressive symptom severity. The Pittsburgh Sleep Quality Index is a 19-item self-report questionnaire used to assess sleep quality and disturbances. Early life stress will be assessed using the Childhood Experience of Care and Abuse questionnaire to determine the effects of maltreatment and upbringing on treatment response and MDD biomarkers.

Participants will also use a personal log to track any newly emerging GI symptoms since the beginning of treatment to assess their tolerability to treatment and keep track of their mood symptoms and sleep. Tolerability and GI effects will be assessed using the Toronto Side Effects Scale and the GI Symptom Rating Scale during all treatment-related visits.

#### Molecular Analysis

Stool samples will be collected and analyzed using 16S ribosomal RNA sequencing at the University of Guelph. These data will be used to assess the relative abundance of microbial species, level of engraftment of MET-2 species, and α and β diversity of the gut microbiome. Urine samples will be collected to analyze soluble metabolites at the University of Guelph, whereas blood samples will be collected for clinical chemistry, hematology, and additional biomarker testing for safety and discovery purposes at Life Labs and Queen’s University, respectively. Statistical analyses will be performed to assess the relationship between immune biomarkers and clinical symptom improvement. Additional safety blood samples may be required in the event of abnormal values or analysis failure. The blood samples collected for safety laboratory analysis will be destroyed after the analyses have been completed.

The specific parameters being assessed are presented in Table S3 in Multimedia Appendix 1, whereas collection time points can be found in Table S1 in Multimedia Appendix 1.

#### Safety Assessments

The primary safety analysis for this study will include the total number of treatment-emergent adverse events (TEAEs) while also categorizing TEAEs by causality, severity, and seriousness assessments made by the investigator by comparing study drug exposure to placebo.

Trends in safety will also be evaluated for the following assessments: physical examinations, vital signs, laboratory results (hematology, lipid levels, and serum chemistry), pregnancy tests, and discontinuations because of adverse events (AEs).

### Demographic Data and Medical History

At the screening visit, a complete medical history will be compiled for each participant. This history will include the use of antidepressant therapies, GI-related disorders and surgeries, depressive episodes, medical history, baseline signs, and symptoms.

#### Vital Signs

Vital signs, including temperature, blood pressure (systolic and diastolic), pulse, and body weight (using a calibrated weight scale), will be measured at screening, baseline, week 4, and week 8. Blood pressure will be measured with a cuff size appropriate to the participant after the participant has been sitting for 5 minutes.

#### Physical Examination

A complete physical examination will be conducted at the baseline visit. The physical examination will include general appearance, skin, head, ears, eyes, nose, throat, lungs, cardiovascular, abdomen, musculoskeletal and extremities, lymph nodes, and neurological. Other body and organ systems may be examined if clinically relevant.

#### Pregnancy Test

A pregnancy test will be performed at baseline. If pregnancy is discovered in a female participant enrolled in the study before the end of dosing, the study drug will be permanently discontinued, and an end-of-treatment visit will be scheduled. If the pregnancy is discovered in a female participant enrolled in the study after the end of dosing, the participant will continue in the study per protocol. If pregnancy occurs in a male participant’s partner at any time during the study, the pregnancy is to also be reported and followed.

### Adverse Events

#### Overview

An AE is any untoward medical occurrence in a participant administered an investigational treatment, which does not necessarily have a causal relationship with this treatment. An AE can therefore be any unfavorable and unintended sign or symptom temporally associated with the use of the investigational treatment, regardless of whether it is related to the treatment. Each participant will be carefully monitored for the development of AEs during the safety reporting period. Both the frequency and severity of AEs will be collected at the time of consent throughout the study until the 2-week follow-up. AEs will be assessed and recorded at all in-hospital visits or through the phone using questionnaires and probing via discussion; these AEs will be categorized by frequency, severity, and causality [7]. Any clinically relevant abnormalities will be noted by the investigator during the follow-up interviews. All AEs will be recorded in an AE log for each participant. Any instances that lead to serious AEs (SAEs) and any untoward medical occurrences at any dose will be reported to the research ethics board (REB).

A TEAE is an AE that begins during the treatment or worsens a pre-existing medical condition (eg, worsening diarrhea). The treatment period is the period during which a participant receives the investigational treatment.

#### Serious Adverse Events

An SAE is a life-threatening adverse medical event that results in death, requires inpatient hospitalization or prolongation of existing hospitalization, results in persistent or significant disability or incapacity, and results in a congenital anomaly or congenital disability [7]. The study site will document all SAEs that occur (whether related to the study drug) and report them to the sponsor within 24 hours of the site’s first knowledge of the event. The collection period for all SAEs will begin after informed consent is obtained and will end after the procedures for the final study visit have been completed.

All SAEs will be followed until resolution. SAEs that remain ongoing past the participant’s last protocol-specified follow-up visit will be evaluated by the principal investigator and NuBiyota LLC. If both parties agree that the participant’s condition is unlikely to resolve, the principal investigator will determine the follow-up requirement.

#### Reporting of Expedited Safety Reports

A suspected unexpected serious adverse reaction (SUSAR) is an SAE that occurs in a participant, the nature or severity of which is not expected per the applicable product information (ie, the investigator’s brochure).

The sponsor and investigator will report any SUSARs concerning the drug that has occurred in the study to regulatory authorities:

- If it is neither fatal nor life-threatening, within 15 days after becoming aware of the information.
- If it is fatal or life-threatening, within 7 days after becoming aware of the information. The sponsor and investigator shall, within 8 days after having informed the REB and Health Canada of a fatal or life-threatening SUSAR, submit a complete follow-up report in respect of that information that includes an assessment of the importance and implication of any findings made.

### Statistics

#### Sample Size Determination

The sample size for this clinical investigation was calculated based on the results from the pilot study and the change from baseline to week 8 of the first 13 participants for the MADRS (primary endpoint) and GAD-7 scores (first secondary endpoint). The change from baseline to week 8 was used for the two endpoints, mirroring the time frame for the evaluation of the primary and first secondary endpoints for this clinical investigation. Two series of estimates were generated: change from baseline for the participants with scores at baseline and week 8, and a second imputed data set for participants who did not have a week 8 value using the last observation carried forward. Although the final analysis from this study will use multiple imputation to address missing data, using the last recorded observation results in a higher SD and is considered a reasonable approach to derive a range of estimates. Estimates from the pilot investigation are presented in Table S4 in Multimedia Appendix 1.

For estimation purposes, it is assumed that the placebo participants will have a response that is 50% of the active treatment group, and the SD will be the same between the two treatment groups. The randomization will be in a 1:1 ratio (active:placebo), and the type 1 error rate will be 5%. The resulting sample sizes for 80%, 85%, and 90% power are presented in Table S5 in Multimedia Appendix 1.

If the true mean difference between the active and placebo groups is the midpoint between the observed and imputed results, a minimum target sample size of 50 participants will be required for the primary endpoint (MADRS: change at 8 weeks) and 79 participants (GAD-7: change at 8 weeks) for the other endpoints. On the basis of these estimates, the maximum number of participants to be enrolled in this clinical investigation is 80, and the target sample size is 60 participants. An interim assessment will be performed after 30 participants have completed the 8-week evaluation or withdrawn prematurely.

#### Populations for Analyses

##### Intent-to-Treat Population

This population includes all participants who consent to participate, meet the inclusion criteria, and are randomized. All baseline characteristics will be summarized based on intent-to-treat (ITT). Participants in the ITT population will be analyzed according to the original treatment assignment, regardless of the actual treatment received. All baseline tables will be based on the ITT population. The ITT population will be the primary population for efficacy endpoints.

##### Per-Protocol Population

The per-protocol population is a subset of the safety population. It includes all participants who meet all of the following criteria:

- No major deviations from protocol eligibility criteria;
- ≥85% of compliance with the treatment schedule; and
- ≥85% of compliance with all study visits where the samples will be obtained and the testing will be performed.

The per-protocol population will be used for the sensitivity analyses of the efficacy endpoints.

##### Safety Population

The safety analysis population will contain all participants who receive at least one dose of study medication (active or placebo). All safety tables will be based on the safety population.

### Statistical Analysis

The statistical analysis plan will be finalized before database lock and will include a more technical and detailed description of the statistical analyses described in this section. This section summarizes the planned endpoints and statistical analyses. Participants who return after the initial treatment but later withdraw from the study will have their final scores for primary outcomes projected to week 8. If data are missing, the data from the last time point will be projected forward.

Changes from baseline to endpoint in scores on the MADRS, HAM-A, CGI, SHAPS, QIDS-SR16, GAD-7, and Pittsburgh Sleep Quality Index will be analyzed using two-tailed *t* tests or analysis of variance between and within the placebo and active arms. All statistical tests will be performed using the statistical program IBM SPSS with a significance level of .05. Similarly, stool samples will be analyzed for their diversity scores using a paired *t* test and repeated measures analysis of variance. Any changes in diversity scores will then be compared with the clinical scores to determine any correlations.

### Direct Access to Source Data or Documents

The investigator or institution shall provide direct access to source data or documents for study-related monitoring, audits, REB review, and regulatory inspection.

### Quality Control and Quality Assurance

#### Study Conduct

This study will be conducted in compliance with the current International Council for Harmonization of Technical Requirements for Pharmaceuticals for Human Use (ICH) Good Clinical Practice (GCP) guidelines. The sponsor shall ensure that all sites have the necessary standard operating procedures to ensure that the study is conducted and data are generated, documented, and reported in compliance with the protocol, ICH GCP, and applicable regulatory requirements. The investigator may not deviate from the protocol without a formal protocol amendment being established and approved by an appropriate competent authority and REB, except when necessary, to eliminate immediate hazards to the participant or when the changes involve only logistical or administrative aspects of the study. Any deviations may result in the participant being withdrawn from the study and render the participant nonevaluable. The sponsor is responsible for the distribution of protocol amendments to the principal investigators and those concerned with the conduct of the study. The principal investigator is responsible for the distribution of all amendments to the REB and all the staff concerned at their center.

#### Study Monitoring

All monitoring visits will be conducted by PhaseAdvance, a contract research organization appointed by NuBiyota LLC, to ensure compliance with the ICH guidelines for GCP (E6). The study monitors will conduct an initiation site visit to the institution to review the protocol and its requirements with the investigators, inspect the drug storage area, and fully inform the investigator of their responsibilities and the procedures for assuring adequate and correct documentation. During the study, the monitor will make regular site visits to review protocol compliance and individual participants’ medical records and ensure that the study is being conducted according to pertinent regulatory requirements. The review of medical records will be conducted in a manner that ensures confidentiality is maintained. No information in these records regarding the identity of the participants will leave the study center. Sponsor monitoring standards require full verification of the presence of the signed informed consent form (ICF), adherence to the inclusion and exclusion criteria, documentation of SAEs, and recording primary efficacy and safety variables. The clinical research associate will review source data compared with the case report forms (CRFs) and verify source data according to the study-specific monitoring plan. The study design, frequency of participant visits, and site enrollment rate will determine the frequency of monitoring visits. Upon study completion, the clinical research associate will visit the site to conduct a study termination visit, which will include the collection of any outstanding documentation.

### Ethics

#### Written Informed Consent

To obtain and document informed consent, the investigator will comply with the applicable regulatory requirements and adhere to ICH GCP and the ethical principles that have their origin in the Declaration of Helsinki.

Informed consent shall be documented using a written consent form approved by the REB. The ICF must be signed and dated by the participant and investigator (or designated research professional) before protocol-specific procedures (screening or treatment) are performed. A consent form template will be provided by the sponsor and adapted by the investigator to meet the center, state, and country ethical guidelines, as appropriate.

Participants will be given a copy of the fully executed consent form, and the original will be maintained with the participant’s records.

#### Research Ethics Board

This study will not commence until review and approval of the study protocol within each site and ICF has been obtained by local REBs. Any amendments to the study will be submitted concurrently to all REBs for review and approval unless they are required to eliminate immediate hazards to study participants.

### Data Handling and Record Keeping

#### Data Collection

Clinical data will be collected by the study coordinator as a source document and transcribed into REDCap (Research Electronic Data Capture), a web-based data collection software. REDCap will only contain the participant’s ID but no personal data.

#### Retention of Records

Essential documents, as defined by ICH GCP, include the signed protocol and any amendments, copies of the completed CRFs (for site archiving, digital versions of e-CRF data for specific participants will be provided), signed ICFs, hospital records and other source documents, REB approvals and all related correspondence including approved documents, drug accountability records, study correspondence, and a list of participants’ names and addresses. Essential documents should be retained for at least 5 years.

## Results

This study was approved by the Queen’s University Health Sciences and Affiliated Teaching Hospitals REB on September 2, 2020. Owing to COVID-19–related delays, participant recruitment did not start until March 2021. Given the association between the GBA and symptoms of depression, it is expected that participants who receive MET-2 will have a significant reduction in depressive symptoms as determined by the MADRS compared with participants who received the placebo. This will be assessed by comparing pre- and posttreatment scores.

## Discussion

### Optimal Treatment Methods

With MDD affecting 3.7 million Canadians throughout their lifetime [28], there is a pressing need to find optimal treatment methods for these individuals. Although there are numerous psychological, pharmacological, and neurostimulation treatment options available for people experiencing MDD, it is challenging to find personalized treatments. With high individual variability in factors affecting the illness and presentation of symptoms, an effective treatment may not be effective for another [12]. Given these circumstances, novel treatment methods are being developed and evaluated for their ability to alleviate the symptoms of depression. One of these novel treatment approaches involves assessing individuals’ gut health and targeting the gut microbiome through the GBA [7,12].

The gut plays a critical role in modulating physiological processes of the body, such as the immune system and GI functioning, while also playing a role in regulating aspects of brain development [13,29]. Although there has been a growing recognition of the GBA in the last few years, further research is warranted to fully elucidate the underlying mechanisms affecting mood and gut health. Fortunately, if MET-2 can alleviate symptoms of depression and produce a lasting effect on mood both during and after treatment, it may provide promising results for microbe therapy as a treatment method and may help explain how the GBA relates to mood. Similarly, given the adaptable nature of microbiota and how it can be influenced by external factors, the microbiome may indicate each individual’s predicted response to treatment, severity, and course of MDD.

### Limitations

Limitations to the many studies examining the efficacy of GBA treatments include small sample sizes and thus less generalizable results. Hence, in this trial, we have addressed these concerns by designing a multisite, double-blind, randomized controlled trial. We also hope to enroll 60 participants to determine any significant trends between changes in mood and MET-2 treatment. To our knowledge, assessing the effects of a daily, orally administered microbial therapeutic product such as MET-2 on mood, at this level of scale, has never been performed before. This study would not only be the first of its kind to highlight the potential capabilities of microbe therapy in treating symptoms of depression but also to expand the scope of pre-existing literature surrounding the GBA and how it mediates mood and behavior.

## Appendix

Multimedia Appendix 1 Supplementary tables and textboxes.

Multimedia Appendix 2 Peer-review report.

## Abbreviations

AE: adverse event
CGI: Clinical Global Impressions
CRF: case report form
FMT: fecal microbiota transplantation
GAD: generalized anxiety disorder
GBA: gut-brain axis
GCP: Good Clinical Practice
GI: gastrointestinal
HAM-A: Hamilton anxiety rating scale
ICF: informed consent form
ICH: International Council for Harmonization of Technical Requirements for Pharmaceuticals for Human Use
Ig: immunoglobulin
ITT: intent-to-treat
MADRS: Montgomery-Asberg Depression Rating Scale
MDD: major depressive disorder
MET: Microbial Ecosystem Therapeutic
QIDS: quick inventory of depressive symptomatology
REB: research ethics board
REDCap: Research Electronic Data Capture
SAE: serious adverse effect
SHAPS: Snaith-Hamilton pleasure scale
SUSAR: suspected unexpected serious adverse reaction
TEAE: treatment-emergent adverse event
