# Supplementary material for: The Safety and Efficacy of Microbial Ecosystem Therapeutic-2 in People With Major Depression: Protocol for a Phase 2, Double-Blind, Placebo-Controlled Study
Source: JMIR Res Protoc. 2021 Sep 22;10(9):e31439. doi: 10.2196/31439 (PMC8495575; doi:10.2196/31439)
Supplement: Multimedia Appendix 1 [file resprot_v10i9e31439_app1.pdf]

This is a Multimedia Appendix to a full manuscript published in the J Med Internet Res. For full copyright and citation information see http://dx.doi.org/10.2196/jmir.31439

Multimedia Appendix

Table S1: Schedule of assessments

Procedures	Study Timeline					
	Screening[a]	Week 0	Week 2	Week 4	Week 6	Week 8
Study Criteria and ICF Review and Signing	X					
MINI[b] Diagnostic Interview	X					
Demographics	X					
Medical and Antidepressant History	X					
Physical Examination		X				
Vital Signs	X	X	X	X	X	X
Pregnancy Test (Females)		X				
MADRS[c]	X	X	X	X	X	X
HAM-A[d]		X	X	X	X	X
GAD-7[e]		X	X	X	X	X
CGI[f]		X	X	X	X	X
SHAPS[g]		X	X	X	X	
QIDS-SR16[h]		X	X	X	X	
PSQI[i]		X	X	X	X	
TSES[j]		X	X	X	X	
GSRS[k]		X	X	X	X	
CECA[l]					X	
AE[m] Monitoring		X	X	X	X	X
Blood Samples Collection	X			X		X
Urine Sample Collection		X		X		X
Stool Specimen Collection		X	X	X	X	X

[a] Time between screening to baseline can be up to 10 days.
[b] Mini-International Neuropsychiatric Interview
[c] Montgomery-Asberg Depression Rating Scale
[d] Hamilton Anxiety Rating Scale
[e] Generalized Anxiety Disorder Scale
[f] Clinical Global Impressions Scale
[g] Snaith-Hamilton Pleasure Scale
[h] Quick Inventory of Depressive Symptomatology
[i] Pittsburgh Sleep Quality Index
[j] Toronto Side Effects Scale
[k] Gastrointestinal Symptom Rating Scale
[l] Childhood Experience of Care and Abuse Questionnaire
[m] Adverse Event

This is a Multimedia Appendix to a full manuscript published in the J Med Internet Res. For full copyright and citation information see http://dx.doi.org/10.2196/jmir.31439

Table S2: MET-2 dosing

Dosing	Treatment Period		
	Baseline to Week 2	Weeks 2 to 4	Weeks 4 to 6
Loading/Booster Dose (10 capsules or 5.0 g/day of MET-2 or placebo[a])	Days 1 and 2 only	Days 15 and 16 only	NR[d]: Days 29 and 30 only[b]
Maintenance Dose (3 capsules or 1.5 g/day MET-2 or placebo)	Days 3-14	Days 17-28	R[c]:Days 29-42<br>NR[d]: Days 31-42

[a] The present study involves 6 weeks of regular maintenance dose with a 2-day loading dose/booster at baseline and Week 2, during which period participants will not take the maintenance dose.
[b] Only Non-Responders will receive the booster dose at this point (Week 4).
[c] Responders
[d] Non-Responders

Table S3: Laboratory parameters

Sample Collected		Timepoints		
		Screening	Week 4	Week 8
**Serum Chemistry**				
	Albumin	x		x
	ALT[a]	x		x
	ALP[b]	x		x
	AST[c]	x		x
	Carbon Dioxide	x		x
	Chloride	x		x
	Creatinine	x		x
	Glucose	x		x
	Potassium	x		
	Sodium	x		x
**Hematology**				
	Hematocrit	x		x
	Hemoglobin	x		x
	Platelet count	x		x
	Red blood cell count	x		x
	White blood cell count and differential (neutrophils, eosinophils, basophils, lymphocytes, and monocytes)	x		x
**Additional Biomarkers**				

This is a Multimedia Appendix to a full manuscript published in the J Med Internet Res. For full copyright and citation information see http://dx.doi.org/10.2196/jmir.31439

	IL-10[d]	x	x	x
	IL-6[d]	x	x	x
	CRP[e]	x	x	x
	TGF- β[f]	x	x	x
	TNF-α[g]	x	x	x
	IgA[h]	x		x
	IgG[h]	x		x
	IgM[h]	x		x
	Triglycerides	x		x
	HDL[i]	x		x
	LDL[j]	x		x
	Lipid Ratio Cholesterol	x		x

[a] alanine aminotransferase

[b] alkaline phosphatase

[c] aspartate aminotransferase

[d] interleukin

[e] c-reactive protein

[f] transforming growth factor

[g] tumour necrosis factor

[h] immunoglobulin

[i] high-density lipoprotein

[j] low-density lipoprotein

Table S4: Sample size estimates

Parameter	Number of Participants	Week 8 Mean +/- Standard Deviation Change (Week 6 minus Baseline)
MADRS (observed)	7	-10.29 +/- 4.23
MADRS (imputed)	11	-8.36 +/- 6.33
GAD-7 (observed)	7	-8.00 +/- 5.03
GAD-7 (imputed)	11	-5.82 +/- 5.27

Table S5: Power estimates

Parameter	80% Power	85% power	90% power
MADRS (observed)	24	28	32
MADRS (imputed)	76	86	100
GAD-7 (observed)	52	60	70
GAD-7 (imputed)	106	120	140

This is a Multimedia Appendix to a full manuscript published in the J Med Internet Res. For full copyright and citation information see http://dx.doi.org/10.2196/jmir.31439

Textbox S1: Inclusion/Exclusion Criteria

Inclusion Criteria

1. 18-45 years of age and able to provide informed consent.
2. Willing to participate in follow up as part of the study
3. Diagnosis of MDD as determined by the MINI
4. Current depressive episode with a MADRS score of ≥15.
5. Able to understand and comply with the requirements of the study
6. Able to provide stool, urine, and blood samples.

Exclusion Criteria

1. History of chronic diarrhea
2. Need for regular use of agents that affect GI motility (narcotics such as codeine or morphine, agents such as loperamide or metoclopramide)
3. Colostomy
4. Elective surgery that will require preoperative antibiotics planned within 6 months of enrolment
5. History of bariatric surgery.
6. Pregnant, breastfeeding, or planning to get pregnant in the next 6 months
7. Any condition for which, in the opinion of the investigator, the participant should be excluded from the study.
8. Current use of any antidepressant/antianxiety drug (eligible to participate after a 4-week washout period)
9. Currently participating in a structured psychotherapy program
10. More than three depressive episodes throughout lifetime
11. Having failed an antidepressant or psychotherapeutic treatment during current depressive episode
12. Use of any antibiotic drug in the past 4 weeks (may be eligible to participate after a 1-month washout period)
13. History of alcohol or substance dependence in the past 6 months
14. Daily use of probiotic product in the past 2 weeks (may be eligible to participate after a 2-week washout period)
15. Use of any type of laxative in the last 2 weeks.
16. Consumption of products fortified in probiotics (may be eligible to participate after a 2-week washout period)
17. High suicidal risk, as measured by MADRS item 10 score equal to or higher than 4.
18. Current psychotic symptoms
19. Diagnosed with bipolar disorder
20. History of epilepsy or uncontrolled seizures
21. Immunodeficiency (immuno-compromised and immuno-suppressed participants; e.g., acquired immune deficiency syndrome [AIDS], lymphoma, participants undergoing long-term corticosteroid treatment, chemotherapy and allograft participants)
22. Unstable medical conditions or serious diseases/conditions (e.g. cancer, cardiovascular, renal, lung, diabetes, psychiatric illness, bleeding disorders, etc.)
23. The use of natural health products (e.g., St. John's Wort, passion flower, etc.) that affect depression
