# Supplementary material for: The Safety and Efficacy of Microbial Ecosystem Therapeutic-2 in People With Major Depression: Protocol for a Phase 2, Double-Blind, Placebo-Controlled Study
Source: JMIR Res Protoc. 2021 Sep 22;10(9):e31439. doi: 10.2196/31439 (PMC8495575; doi:10.2196/31439)
Supplement: Multimedia Appendix 2 [file resprot_v10i9e31439_app2.pdf]

January 18, 2021

Application Ref. IT23035

RE: Mitacs Accelerate Proposal

Project Title:	A Phase 2, Double-blind, Placebo-controlled Study of the Safety and Efficacy of Microbial Ecosystem Therapeutic-2 (MET-2) in Patients with Major Depression
Internship Supervisor(s):	Roumen Milev
Intern(s):	Cassandra Sgarbossa, Arthi Chinna Meyyappan
Department:	Department of Psychiatry
Institution:	Queen's University
Partner Organization:	NuBiyota

Dear applicants,

Your application to Mitacs Accelerate has successfully passed research review. Your research project has been approved for a total grant of $180,000.00 that will be delivered through eligible internships. Your internship should not start prior to confirmation of eligibility as outlined below:

1. Mitacs must receive partner funds before each internship start date to pay the intern(s) on time;
2. Project leads must confirm intern name(s) and start date(s). No costs for an internship can be incurred prior to the research project approval and the receipt of partner funds for the internship;
3. To identify a new intern, the Intern Profile form (including CV and memorandum) must be submitted to Mitacs before the applicable internship's start date and obtain Mitacs approval;
4. Project leads must obtain the following certification(s) or approval(s) before the project begins: University Research Ethics Board approval and Biohazard approval.

All awards are subject to ongoing program eligibility, continuation of funding by the partner organization(s), and continuation of funding by our government partners. Mitacs Accelerate gratefully acknowledges the financial support of the Government of Canada and the Province of Ontario through the Ministry of Advanced Education and Skills Development.

Welcome to Mitacs Accelerate. With this program, interns can apply their specialized expertise while companies gain a competitive advantage. We look forward to the success of your project.

If you have any questions, please contact Deirdre Sequeira, Grant Management Specialist, at dsequeira@mitacs.ca.

Sincerely,

R. Ben Mrad

Ridha Ben Mrad
Chief Research Officer and Associate Academic Director

Enclosures:
- Appendix A: Referee comments
- Appendix B: Terms and Conditions of the Award
- Attachment: Intern Onboarding Information

Appendix A: Referee Comments

Your proposal has undergone external review. We received two review reports and the comments are in general positive. The reviewers are supportive of your research rationale, objectives and methodological approach. Please note that the reviewers would have appreciated a few more experimental details and raise specific limitations that we invite you to consider to strengthen your project (see responses to question #4).
In particular, we'd like to notify that experimental approaches and analyses described in your proposal do not seem to integrate potential differences related to age, sex and gender. Given the nature of your project, the data to be collected and as it is an emerging critical factor in translational research, we'd like to encourage you to integrate these variables in your approaches. As we believe that this question may be of great interest to your research question, please consider of incorporating this issue into your study design and analyses in order to strengthen the value of your results as well as the impact of your conclusions. For more information, please visit the following site from CIHR https://cihr-irsc.gc.ca/e/8673.html.

The comments are provided below for your information. We wish the applicants success in their research.

We expect that you and your collaborating researchers will follow the directives provided by applicable institutional, municipal, provincial/territorial, and federal bodies in relation to the COVID-19 pandemic. For example, mitigation strategies may be required to accommodate requirements for physical distancing and limitations on gathering size.

Reviewer 1

1) Mitacs Accelerate supports research-based internships. Does the proposed project qualify as research in its discipline?
The proposed project qualifies as research in the discipline specified in the proposal (Life Sciences). It is also aligned with the academic disciplines in which the two proposed interns have been or are currently trained (Neuroscience, Psychology, Life Sciences). This is a second (ongoing?) phase of a proposal that I have evaluated about a year ago so I am assuming that the initial steps of safety, efficacy, and tolerability of the MET-2 product are still undergoing validation (perhaps due to covid-19 research interruptions). Again, this is a very promising project and findings from this research may provide new opportunities of alternative/adjunctive treatment in people with depression and/or anxiety.

2) Is the project appropriate for the academic degree level of the intern(s)?
The proposed project is appropriate for an intern at the PhD level, especially as the intern (A.C. Meyyappan) was involved in the first phase of the study and is thus likely well trained in the study procedures. I see that a second intern at the MSc level will be involved in the study (C. Sgarbossa), which will certainly help with the challenge of participant recruitment and the several types of measures involved (clinical and molecular). The fact that the industrial partner will provide space and expertise to learn how to analyze blood and stool sample analyses (page 12; i, 2, a) will also help the interns face some of these challenges.

3) Are the objectives clear?
The main objective of this proposal is to assess changes in mood and anxiety symptoms in individuals with depression throughout a 6-week treatment with MET-2, a compound composed of 40 strains of freeze-dried bacteria isolated and purified from a 25-year old healthy donor (developed by the industrial partner NuBiyota). Secondary objectives will examine (1) changes in metabolic functioning/levels, (2) safety and tolerability, and (3) potential correlations between early life stress and changes in MDD biomarkers and response to treatment. These objectives are clear.

4) Is the methodology appropriate to achieve the objectives?
The methodology described to conduct the proposed research project appears appropriate. The MINI is one of the gold standards to identify neuropsychiatric disorders (here major depression), the study questionnaires to assess depression /anxiety symptoms are adequate (e.g., MADRS, GAD-7) as well as the scales for sleep quality and early-life stress. However I am wondering why so many scales are used to determine depression and anxiety severity - isn't there a risk that this will be very demanding for this clinical population?

The use of the 16S assay to assess fecal microbiota composition (and thus microbial engraftment between the donor and participants) is appropriate. Unfortunately, as in the initial proposal, no details are provided on the sample collection procedures, which are important in the context of the current proposal as it appears that proteomic and metabolic analyses will be conducted (page 7, section 2.5). As well, it is specified that "All except for inflammatory markers and immunoglobulin levels will be assessed locally at each site; those will be stored for later, central, analyses" (page 9). I understand from this that analyses of inflammatory factors will be completed by the industrial partner (or by the intern under the guidance of the partner) but what about the other factors (lipids, glucose, etc) - these will be assayed in house at Queens? Although the methodology in general appears adequate, and I trust that analyses will be conducted adequately, I feel more details should have been provided to be in a better position to assess if all aspects of the methodology are appropriate to achieve the

objectives.

5) Is the timeline realistic?
The 2-year timeline for recruiting 80 unmedicated participants with major depression is somewhat narrow considering all the inclusion/exclusion criteria (which are admittedly necessary). The fact that data will be analyzed "as data is collected" could be a way to overcome this limitation but I think this may be challenging to complete recruitment in the proposed 2-year time frame. Perhaps it should be considered to allow recruitment during the 8-month period (months 24-32) planned for data analysis only.

6) Other comments and suggestions for the applicants. For example, is there prior work that should be considered by the researchers?
None.

Reviewer 2

1) Mitacs Accelerate supports research-based internships. Does the proposed project qualify as research in its discipline?
Yes. This study examines the effect of a gut microbial treatment on symptoms of depression. This is a double-blind, placebo controlled study.

2) Is the project appropriate for the academic degree level of the intern(s)?
I believe so. The application includes two interns, a PhD student and a Master's student. While the methodology is well described,. However, the methodology is appropriate and the Academic Supervisor has the breadth of experience to provide a suitable training environment.

3) Are the objectives clear?
Yes. The primary objective of this study is to evaluate the effectiveness of a novel gut microbial-targeted therapeutic (microbial ecosystem therapeutic 2; MET-2) on objective changes in mood and anxiety in patients diagnosed with major depression disorder or generalized anxiety disorder. Secondary objectives include assessing the changes in metabolic function following treatment, assessing the safety and tolerability of this treatment, and assessing the correlation of early life stress with differences in biomarkers for depression and treatment responsivity.

4) Is the methodology appropriate to achieve the objectives?
Yes. This is a well designed placebo-controlled double blinded study. Exclusion and Inclusion criteria are well defined. A limitation is that subjective measures of diet or nutrient intake are not collected. This would be useful to determine underlying differences in gut microbiota and treatment responsivity. Also, it is not clearly stated that placebo and treatment arms will be age- and sex-matched.

5) Is the timeline realistic?
Yes, the timeline is appropriate to complete this study.

6) Other comments and suggestions for the applicants. For example, is there prior work that should be considered by the researchers?
None.

Appendix B: Mitacs Accelerate - Terms and Conditions of the Award

Please read all conditions carefully. Any questions or requests for clarification should be directed to your Grant Management Specialist listed in the letter above.

1. General guidelines:

1.1.The average amount of partner interaction for each four-month internship across the project should be 50% (or minimum of 25% with sufficient justification in original application).

1.2.The maximum allowable number of Accelerate internships varies depending on the intern's academic designation:
- 1.2.1. College students can complete one internship unit;
- 1.2.2. Master's students can complete up to four internship units;
- 1.2.3. PhD students can complete up to eight internships units; and,
- 1.2.4. Postdoctoral fellows can complete up to nine internships units.

1.3.The start date of each internship cannot predate the research approval of the project and the receipt of partner funds for the internship.

1.4.Awarded funds must be allocated according to the internship period(s) and stipend/salary allocations, which will be outlined on the Mitacs Award Letter.

1.5.Please report any substantial change (internship dates, project personnel, partner organizations, etc.) concerning your project immediately to your Mitacs Grant Management Specialist contact.

2. Funding:

2.1. Contribution funds from the partner organization must be paid to Mitacs prior to the internship start date to facilitate efficient payment process for interns.

2.2. Funding will not be released for un-named interns.

2.3. The stipend portion of the award must be spent as outlined in the award letter. No extensions can be granted on stipend amounts.

2.4. The research grant portion of the award must be spent by end date of the award. Any remaining research funds may be used by the academic supervisor to supplement the intern's stipend at the Academic Supervisor's discretion. Any funds not spent by this date must be returned to Mitacs unless an extension of the award is granted by Mitacs. To obtain an extension, the supervisor needs to contact Mitacs before the end date of the award, with justification of such a request.

2.5. In-kind contributions cannot be used towards matching Mitacs funds.

2.6. Inter-university transfers are not permitted.

2.7. To ensure that your project complies with the Mitacs university agreement and federal granting council policies, you must provide Mitacs with copies of all contracts that govern the research being performed using Mitacs funds.

3. Intern Stipends:

3.1. Each intern must receive a minimum $10,000 stipend/salary for each internship unit.

3.2. All stipend/salary funds must be paid over a period of no less than 4 months and no greater than 6 months per internship unit.

4. Research Funds:

4.1. The remaining funding may be used to cover expenses in support of the internship research and its related costs, as directed by Tri-Council.

4.2. Relevant research expenses incurred outside of the indicated funding period may be approved by Mitacs on a case-by-case basis.

5. Reporting:

5.1. If the award straddles fiscal years, the University will provide a separate Form 300 (statement of account) for each account by May 31st for each fiscal year until the account reaches a zero balance. The University will provide a Final Form 300 for each account within 30 days of the end date of this award. The Academic Supervisor and Finance Officer must sign the Form 300s and forward to Mitacs once finalized. Please include the Application Reference number and Project Title on the Form 300 and submit to form300@mitacs.ca

5.2. Projects involving more than 50 internship units and spanning longer than 2 years are required to submit a progress report to Mitacs at the mid-point of the project. Please designate one academic supervisor to be the coordinator who is responsible for submitting the report to Mitacs. Failure to submit a satisfactory report may result in delay or cancellation of subsequent funding.

5.3. All parties are required to provide Mitacs with a final report and completed exit surveys at the end of the Mitacs Accelerate internship.

Exit Survey links will be emailed to students after the end of their internship, and to partners and professors after the end of the project.

The link to the Final Report is listed below:
http://www.mitacs.ca/sites/default/files/uploads/page/mitacs_accelerate_final_report_mar2013.zip

6. Acknowledgement of Mitacs:

6.1. All Mitacs-sponsored results must explicitly credit Mitacs. This includes work by professors, post-docs, interns, and research associates. Mitacs funding can be acknowledged in publications using the following statement: "This work was supported by Mitacs through the Mitacs Accelerate Program." All networking activities initiated by your project for which Mitacs funds are used must also credit Mitacs. For advertising purposes, the Mitacs logo is available in several formats for download from the website at www.mitacs.ca
